# Epidemiology of snake bites in selected areas of Kenya

**DOI:** 10.11604/pamj.2018.29.217.15366

**Published:** 2018-04-20

**Authors:** Francis Okumu Ochola, Mitchel Otieno Okumu, Gerald Mwangi Muchemi, James Mucunu Mbaria, Joseph Kangangi Gikunju

**Affiliations:** 1 Department of Pharmacology and Toxicology, School of Medicine, Moi University, Eldoret, Kenya; 2 Department of Pharmacy, Jaramogi Oginga Odinga Teaching and Referral Hospital, Kisumu, Kenya; 3 Department of Public Health, Pharmacology & Toxicology, Faculty of Veterinary Medicine, University of Nairobi, Nairobi, Kenya; 4 Department of Medical Laboratory Science, College of Health Sciences, Jomo Kenyatta University of Agriculture and Technology, Nairobi, Kenya

**Keywords:** Snake bite, neglected emergency, Kenya, epidemiology, snake antivenom

## Abstract

**Introduction:**

Snake bites are a silent public health problem in Kenya. Previous studies on snake bites in the country have mainly focused on identifying offending snake species, assessing the severity of envenomation and testing the efficacy of antivenom. Factors associated with snake bites in the country are yet to be fully understood. The aim of this work was to determine pharmaco-epidemiological factors associated with snake bites in areas of Kenya where incidence, severity and species responsible for snake bites have been reported.

**Methods:**

Kakamega provincial hospital, Kabarnet, Kapenguria and, Makueni district hospitals were selected as study sites based on previous findings on incidence, severity and species responsible for snake bites in catchment areas of these hospitals. Persistent newspaper reports of snake bites in these areas and distribution of snakes in Kenya were also considered. Cases of snake bites reported between 2007-2009 were retrospectively reviewed and data on incidence, age, site of the bites, time of bite and antivenom use was collected.

**Results:**

176 bites were captured, 91 of which occurred in 2009. Individual incidence was between 2.7/100,000/year and 6.7/100,000/year. Bites peaked in the 1-15 year age group while 132/176 bites were in the lower limb area and 49/176 victims received antivenom. Most bites occurred during the dry season, in the bush and in the evening. Overall mortality was 2.27%.

**Conclusion:**

There is a need to sensitize the Kenyan public and healthcare personnel on preventive measures, first aid and treatment of snake bites.

## Introduction

Snake bites are a neglected emergency in Kenya. This is because there is low awareness of snake bites as a public health problem in the country. Few studies have been carried out to evaluate the magnitude of the problem of snake bites in Kenya. Coombs and co-workers reported the incidence of snake bites in Kakamega and Western Kenya, Lake Baringo and Laikipia, Kilifi and Malindi as well as Northern Kenya to be between 1.9/100,000/year and 67.9/100,000/year [1]. Additionally, they reported that the mortality rate of snake bites in these areas was 0.45/100,000/year [1]. Furthermore, Kihiko reported that snake bites at Kitui District hospital were majorly characterized by compartment syndrome and focal gangrene [2]. Puff adders, black spitting cobras, black mambas and the boomslang have been reported to be behind a majority of the snake bites in Kenya [3-6]. Recently, Harrison and others reported that some of the snake antivenoms in Kenya were not pre-clinically effective against the medically important snakes in the country [7]. However, there is a paucity of information on the pharmaco-epidemiological factors associated with snake bites in Kenya. The aim of the present study was to determine the pharmaco-epidemiological factors associated with snake bites in selected areas of Kenya where previous reports of the incidence, severity and species responsible for snake bites have been documented.

## Methods

**Figure 1:**
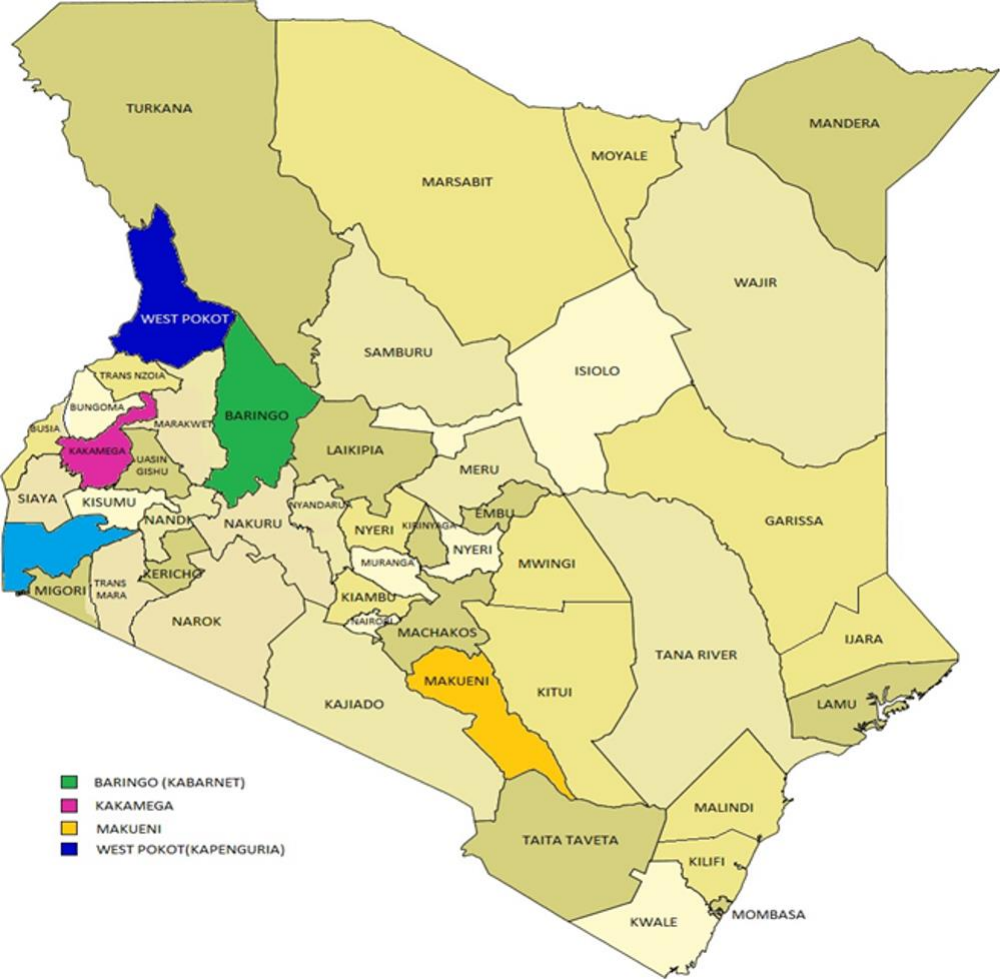
Map of Kenya showing study areas

**Study sites**: A five-point criterion was used in selecting the study sites. I) Findings of a report by Coombs and co-workers on the incidence, severity, and species responsible for snake bites in the catchment areas of the study sites. II) Findings of a report on the distribution of medically important snake species in catchment areas of the study sites [6] (Table 1). III) Multiple newspaper reports of snake bites in catchment areas of the study sites [8-14]. IV) Reports that environmental conditions similar to those experienced in the catchment areas of the study sites favor snake inhabitation [15-17]. V) The fact that the sites were the major health facilities within their catchment areas [18-21]. The selected areas were Kabarnet, Kakamega, Kapenguria and Makueni (Figure 1).

**Table 1:** Distribution of medically important snakes in Kenya

Type of snake	Areas of Distribution
Black mamba (*Dendroaspis Polylepsis*)	Kakamega forest, Lake region, Mau forest, Masai Mara, Lake Naivasha, Aberdares, Mount Kenya, Lake Baringo, Mount Elgon, Kakuma, North Eastern province, Coastal regions, Lamu, Malindi, Mombasa, Ukambani (Makueni)
Eastern Green mamba (*Dendroaspis angusticeps*)	Aberdares, Lake Naivasha, Mount Kenya, Coastal region
Eastern Jameson’s mamba (*Dendroaspis jamesoni*)	Kakamega forest, Lake region, Mau forest, Masai Mara.
Puff adder (*Bitis arietans*)	Same distribution as for Black Mamba
Gaboon viper (*Bitis gabonica*)	Kakamega forest, Lake region, Mau forest, Masai Mara
Rhino viper (*Bitis nasicornis*)	Kakamega forest, lake region, Mau forest, Masai Mara
Saw- scaled viper (*Echis carinatus*)	Lake Baringo, Kakuma, Mount Elgon, Makueni, Tsavo National Park, North Eastern Province.
Black-necked spitting cobra (*Naja nigricollis*)	Distribution as for Black Mamba
Red spitting cobra (*Naja pallida*)	Lake Baringo, Mt. Elgon, Kakuma, North Eastern Province, Ukambani
Large brown spitting cobra (*Naja ashei*)	Kakamega forest, lake region, Maasai mara, Mau forest, Kakuma, Mt. Elgon, Lake Baringo, Aberdares, Mt. Kenya, coastal parts of Kenya
Egyptian cobra (*Naja haje*)	As for Black mamba except it is absent in Coastal regions
Forest cobra (*Naja melanoleuca*)	Kakamega forest, Lake region, Mau forest, Masai Mara, Lake Naivasha, Aberdares, Mt. Kenya, Lake Baringo, Mt. Elgon, Kakuma, Nairobi, Makueni, Tsavo, Coastal region
Boomslang (*Dispholidus typus*)	Distribution as for forest or black and white Cobra
Twig snake (*Thelotornis Kirtlandii*)	Coastal region
Mole Viper, Atractaspis species	Distribution as for Black mamba
Night adders, Causus species	As for Black mamba except North Eastern Province
	
	

Source: www.bioken.com [6]

**Study design**: This was a descriptive study of pharmaco-epidemiological factors responsible for snake bites in the selected areas.

**Data abstraction from hospital records**: Records of all patients with snake bites (ICD-10 system) from January 2007 to December 2009 were reviewed and data on gender, age, number of bites, parts of body bitten, antivenom use and mortality collected.

**Face to face interview of informants**: Semi-structured questionnaires were developed and pre-tested on respondents to check for suitability and validity. Twenty informants (clinicians, nurses in charge of vaccines and pharmacy personnel) were interviewed using the questionnaires which were tailored for each cadre. This was done in order to collect data on different aspects of snake bites. Clinicians were interviewed on the time of bite, the location of the victims at the time of bite, gender, occupation and age, the season of bite and part of the body bitten. They were also interviewed on the period of time taken to seek treatment in hospital, cultural perceptions on snake bites, hospital treatment protocols, availability of antivenom, the effectiveness of antivenom and challenges in the management of snake bites. Nurses were interviewed on the cost and availability of antivenom in the health facilities while personnel in private pharmacies were interviewed on the availability of antivenom in private pharmacies. Permission was sought from the hospital in-charges and ethical approval obtained from the Institutional Ethics Committee (University of Nairobi, J56/72044/08).

**Statistical analysis**: Data was captured using Microsoft Excel (MS Excel) and analyzed using Statistical Software for the Social Sciences (SPSS) version 20. Categorical variables were summarized in percentages and frequencies while continuous variables were represented as the mean and standard deviation.

## Results

**Figure 2:**
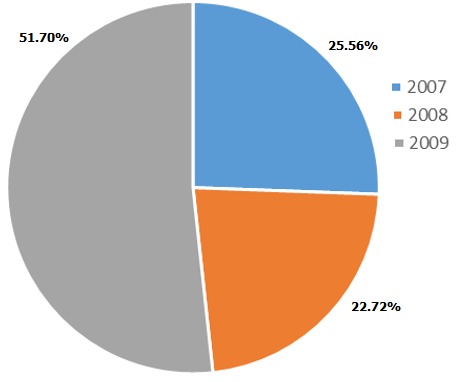
Distribution of snake bites in the study area over a three year period

**Incidence and year wise distribution of bites**: There were a total of 176 bites in all the study sites over the three year study period. 45 cases occurred in 2007, 40 in 2008 and 91 in 2009 (Figure 2). The incidence of snake bites in Kabarnet, Kakamega, Kapenguria and Makueni was 6.7/100,000/year, 4.6/100,000/year, 2.7/100,000/year and 5.4/100,000/year respectively (Table 2).

**Table 2:** An estimate of the incidence of snake bites by district in Kenya

District	Number of snake bite victims	Estimated population in the district	Incidence of snake bite per 100, 000 of population per year
Kabarnet	37	555, 561	6.7
Kakamega	77	1, 660, 651	4.6
Kapenguria	14	512, 690	2.7
Makueni	48	884, 527	5.4

**Distribution of snake bite victims based on gender**: Ninety (90) victims were male while 86 were female. All clinicians interviewed opined that males were more likely to be bitten as compared to females.

**Distribution of snake bite victims based on age**: Most bites (41.48%) occurred in the 1-15 year age group with the least occurrence in the 45 year age group (13.07%) (Table 3). However, 75% (3/4) of clinicians interviewed were of the opinion that the 16-30 year age group was the most bitten followed by the 1-15 year age group. Furthermore, 75% (3/4) of clinicians believed that the least bites occurred in the over 45 year age group.

**Table 3:** Age wise distribution of snake bite victims

Age group	Number of bites	Percentage (%)
0-15	73	41.48%
16-30	56	31.82%
31-45	24	13.64%
Over 45	23	13.07%
Total	176	100%

**Distribution of the site of the bites**: Bites on the lower limbs were the most prominent (132/176) followed by hands (16/176), upper arm (13/176), lower arm (11/176) and other parts (4/176). Moreover, all clinicians interviewed opined that the lower limbs were the most likely part of the body to be bitten.

**Distribution of antivenom use based on the number of bites**: Kapenguria had the least (14/176) number of bites but had the highest rate of antivenom use (71.43%) while Kakamega had the highest number of bites (77/176) but with only 29.87% antivenom use. Moreover, despite Makueni having the second highest number of bites, the rate of antivenom use was only 6.25% (Table 4).

**Table 4:** Summary of antivenom use relative to the number of snake bites in the health facilities

Location	Snakebite victims	Received antivenom	Percentage (%) antivenom administration
Kabarnet	37	13	35.14
Kakamega	77	23	29.87
Kapenguria	14	10	71.43
Makueni	48	3	6.25

**Distribution of snake bite victims based on gender, age and antivenom use**: Gender and age distribution of bite victims who received antivenom was as shown in Table 5. Out of the 49 victims who received antivenom, 28 were female and 21 were male. Of the females who received antivenom, 13 were aged between 1-15, 8 were aged 16-30, 4 were aged 31-45 while 3 were over 45 years of age. Furthermore, of the female victims who did not receive antivenom, 24 were aged 1-15, 19 were aged 16-30, and 9 were aged 31-45 while 6 were aged over 45. Of the males who received antivenom, 9 were aged 1-15, 7 were aged 16-30, 1 was aged 31-45 while 4 were aged over 45 years. Moreover, of the male victims who did not receive antivenom, 27 were aged between 1-15, 22 were aged 16-30, and 10 were aged 31-45 while 10 were aged over 45 years.

**Table 5:** Summary of gender, age and antivenom use in the study area

Gender	Age group	Total patients	AA^1^	NA^2^
Female	1-15	37	13 (35.14%)	24 (64.86%)
	16-30	27	8 (29.63%)	19 (70.37%)
	31-45	13	4 (30.77%)	9 (69.23%)
	Over 45	9	3 (33.33%)	6 (66.67%)
	Total	86	28 (32.56%)	58 (67.44%)
Male	1-15	36	9 (25.00%)	27 (75.00%)
	16-30	29	7 (24.14%)	22 (75.86%)
	31-45	11	1 (9.09%)	10 (90.91%)
	Over 45	14	4 (28.57%)	10 (71.43%)
	Total	90	21 (23.33%)	69 (76.67%)

1 Antivenom administered

2 Antivenom not administered

**Distribution of snake bite victims based on age and antivenom use in individual health facilities**: In Kabarnet, victims in the 1-15 year age group received the most antivenom. Conversely, this age group also registered the highest number of victims who did not receive antivenom. This was replicated in Kakamega and Makueni. However, in Kapenguria, the 16-30 year age group received more antivenom compared to any other group. This group also registered the highest number of victims who did not receive antivenom (Table 6).

**Table 6:** Summary of gender, age and antivenom use in individual facilities

Location	Age group	Total patients	AA^1^	NA^2^
Kabarnet	1-15	20	8(40.00%)	12(60.00%)
	16-30	8	3(37.50%)	5 (62.50%)
	31-45	4	0 (0.00%)	4(100.00%)
	Over 45	5	2(40.00%)	3 (60.00%)
	Total	37	13(35.14%)	24(64.86%)
Kakamega	1-15	27	8 (29.63%)	19(70.37%)
	16-30	31	9 (29.03%)	22(70.97%)
	31-45	12	4 (33.33%)	8 (66.67%)
	Over 45	7	2 (28.57%)	5 (71.43%)
	Total	77	23(29.87%)	54(70.13%)
Kapenguria	1-15	7	4 (57.14%)	3 (42.86%)
	16-30	2	2(100.00%)	0 (0.00%)
	31-45	1	1(100.00%)	0 (0.00%)
	Over 45	4	3 (75.00%)	1 (25.00%)
	Total	14	10(71.43%)	4 (28.57%)
Makueni	1-15	19	2 (10.53%)	17(89.47%)
	16-30	15	1 (6.67%)	14(93.33%)
	31-45 Over 45	7 7	0 (0.00%) 0 (0.00%)	7(100.00%) 7 (100%)
	Total	48	3 (6.25%)	45(93.75%)

1 Antivenom administered

2 Antivenom not administered

**Distribution of snake bite victims based on age, part of the body bitten and antivenom use**: Victims bitten on the lower limbs received the most antivenom while those bitten on other parts of the body received the least antivenom (Table 7).

**Table 7:** Summary of the age, part of the body bitten and antivenom use in the health facilities

Part of the body bitten	Age group	Total patients	AA^1^	NA^2^
Hand	1-15	10	2 (20.00%)	8 (80.00%)
	16-30	3	2 (66.67%)	1 (33.33%)
	31-45	1	0 (0.00%)	1 (100%)
	Over 45	2	1 (50.00%)	1 (50.00%)
	Total	16	5 (31.25%)	11(68.75%)
Upper arm	1-15	5	1 (20.00%)	4 (80.00%)
	16-30	4	1 (25.00%)	3 (75.00%)
	31-45	3	0 (0.00%)	3 (100.00%)
	Over 45	1	0 (0.00%)	1 (100.00%)
	Total	13	2 (15.38%)	11(84.62%)
Lower arm	1-15	5	1 (20.00%)	4 (80.00%)
	16-30	3	2 (66.67%)	1 (33.33%)
	31-45	2	2 (100.00 %)	0 (0.00%)
	Over 45	1	1 (100.00%)	0 (0.00%)
	Total	11	6 (54.55%)	5 (45.45%)
Lower limb	1-15	50	17 (34.00%)	33 (66.00%)
	16-30	46	10 (21.74%)	36 (78.26%)
	31-45	17	3 (17.65%)	14 (82.35%)
	Over 45	19	5 (26.32%)	14 (73.68%)
	Total	132	35(26.52%)	96(72.73%)
Other	1-15	3	1 (33.33%)	2 (66.67%)
	16-30	1	0 (0.00%)	1 (100.00%)
	31-45	0	0 (0.00%)	0 (0.00%)
	Over 45	0	0 (0.00%)	0 (0.00%)
	Total	4	1 (25.00%)	3 (75.00%)

1 Antivenom administered

2 Antivenom not administered

**Appraisal of informant age and experience**: The mean age of the informants was ~37 ± 11 years with a mean of 9.8±8.5 years of experience.

### Other factors

**Location, time of the day when the bites occurred and occupation of bite victims**: All clinicians interviewed were in agreement that the bush was the most likely place to be bitten by a snake. Additionally, all informants with the exception of the informant in Kabarnet, considered residential areas to be the least likely place of occurrence of snake bites. Seventy-five percent (3/4) of the clinicians interviewed identified evening as the most likely time for snake bites to occur. All clinicians interviewed were of the opinion that manual laborers were the most likely group of people to be bitten by snakes.

**Season of the year when bites are likely to occur**: All clinicians interviewed considered bites to be higher in the dry season compared to the wet season.

**The period of time taken by victims to seek treatment**: All clinicians interviewed were of the opinion that victims were least likely to report to the hospital within two hours of having been bitten. Fifty percent (2/4) of the clinicians were of the opinion that victims of snake bites were most likely to present to the hospital between 2 and 6 hours after a bite. According to 75% (3/4) of the interviewees, bite victims delayed in presenting to the healthcare facilities owing to the long distances they had to travel to get to the hospital. One interviewee held the belief that poor infrastructure and poverty contributed to delays in seeking treatment for snake bites.

**Cultural beliefs of victims**: Seventy-five percent (3/4) of clinicians held the belief that victims of snake bites sought treatment from traditional healers before visiting the hospital. One interviewee was of the opinion that this observation was due to the easy accessibility of traditional healers. Interviewees in Kapenguria and Makueni ascribed this to the belief that only bewitched people were bitten by snakes. One (25%) of the interviewees opined that culture was the reason behind the use of herbal medications in managing bites.

**Management of snake bites**: Seventy-five percent (3/4) of the clinicians interviewed reported that supportive care followed by the administration of antivenom was the most common course of action adopted in managing snake bites. Supportive care on its own was regarded by 50% of interviewees as the second most likely course of action to be adopted in managing snake bites. The interviewees unanimously agreed that reassurance of the victims was the least considered course of action in managing snake bites. Reassurance was meant to calm anxious patients who were victims of snake bites.

**Availability of antivenom in the hospitals**: All the clinicians interviewed opined that antivenom was not always available in the hospital. Seventy-five percent (3/4) of interviewees attributed this to delays in procurement of antivenom as well as a shortage in supply. However, one of the interviewees attributed the non-availability of antivenom in the hospital to be due to absenteeism by custodians of antivenom in the health facilities. All clinicians interviewed were in agreement that despite antivenom being available sometimes, its use was not always routine. Moreover, the diagnosis that a bite was not from a poisonous snake was unanimously identified as the main reason for the non-usage of antivenom. According to 75% of the interviewees, the occurrence of a bite from a poisonous snake was ruled out if the patient had stayed for more than 24 hours without any symptoms of envenomation. Additionally, 75% (3/4) of the interviewees held the belief that visits by snake bite victims to traditional herbalists were the second most likely reason to hamper the use of antivenom. Seventy-five percent of the interviewees considered side effects as the least likely reason to hamper the use of antivenom.

**Effectiveness of antivenom**: Seventy-five percent of the interviewees were of the opinion that the antivenom available in the health facilities were effective. One interviewee had a dissenting opinion based on her experience of a poor prognosis of snake bites even after the victims had received antivenom.

**Challenges involved in management of snake bites**: All interviewees opined that regular and timely supply of antivenom was the main challenge in the management of snake bite. Seventy-five percent of the interviewees were of the opinion that delayed assessment of patients who qualified for antivenom administration was the second most prevalent challenge they faced.

**Policies that may improve management of snake bites**: Seventy-five percent of clinicians interviewed held the belief that timely supply of antivenom and proper training on management of snake bites would be the most significant policy measures in improving outcomes in the health facilities. One interviewee was of the opinion that urgency in the management of snake bites, timely supply of antivenom and continuous medical education programs would be the most important steps in improving outcomes.

**Availability, diversity, cost of antivenom and referral of patients to purchase antivenom in private pharmacies**: All nurses interviewed were of the opinion that antivenom was available in the health facilities but that there were periods of unavailability. Seventy-five percent of the interviewees reported that antivenom from only one manufacturer was available. One interviewee reported that her facility had antivenom from two different manufacturers. All interviewees reported that antivenom was offered free of charge in the health facilities. 75% of the interviewees were of the opinion that the health facilities did not refer snake bite victims to purchase antivenom from private pharmacies.

**Availability of antivenom in private pharmacies**: It was unanimously agreed amongst the pharmacy personnel interviewed that most private pharmacies did not stock antivenom. Forty-two percent (5/12) of the interviewees attributed the non-availability of antivenom to low demand and the high cost of antivenom to patients while 33% (4/12) of the interviewees attributed the non-availability to be due to low demand only. Moreover, 92% (11/12) of interviewees reported that they received prescriptions from clinicians to supply antivenom to victims of snake bites. One interviewee, however, reported that he had never received any prescription from clinicians to supply antivenom. Ninety-two percent (11/12) of the interviewees were of the opinion that bottlenecks in the procurement of antivenom were a challenge in stocking antivenoms in private pharmacies.

## Discussion

The number of snake bites over the 3 year period was 176 with an incidence of 6.7/100,000/year, 4.6/100,000/year, 2.7/100,000/year and 5.4/100,000/year for Kabarnet, Kakamega, Kapenguria, and Makueni respectively. These estimates were within the range of 1.9/100,000/year and 67.9/100,000/year reported by Coombs and colleagues [1]. However, these estimates were much lower compared to what was reported elsewhere in Africa [22]. The differing incidence of snake bites may be a reflection of the different rates of hospitalization across the catchment areas of these health facilities [22]. Individuals in the 1-15 and 16-30 year age groups were subject to more bites compared to any other age group. Campbell and others reported that snake bites were more prevalent in children than in adults [23]. The curiosity of children, their size, the mobility of their arms and legs while playing and the increased exposure of appendages may prompt snakes to bite children more than adults [24]. Individuals in the 16-30 year age group mainly comprise of adolescents and young adults who are actively involved in herding, gathering firewood, and farming activities. Thus, individuals in this age group may be exposed to snake bites as they perform these duties. Snake bites on the lower limbs accounted for 75% of all cases. Accessibility of the lower limbs to snakes and lack of protective wear such as shoes when walking or working in farms may account for this observation. Snake bites were most likely to occur in the evening and this is consistent with what has been reported by Karunanayake and co-workers [25]. Nocturnal hunting habits of snakes and poor visibility of humans in the evening may account for this observation [26,27]. Snake bites mainly occurred in the dry season. The dry season in Kenya is associated with scarcity of prey and thus victims may have been exposed to bites from snakes in search of water and food. This explanation may be corroborated by newspaper reports on snake bites in these areas [28-31]. Manual laborers were more prone to snake bites than other occupations. This is because these workers were more likely to encounter snakes in their natural habitat. This observation is consistent with the work of Warrell and others [32].

The most number of snake bites occurred in Kapenguria and least in Makueni. Snake bites have been reported to be more prevalent in rural areas of tropical and sub-tropical countries [33]. Kapenguria (1.2482°N, 35.1104°E) is an arid part of Western Kenya inhabited by the pastoralist communities of Turkana and Pokot [34]. The high number of snake bites in Kapenguria may stem from the fact that most inhabitants of this area are pastoralists. Thus, bites may have occurred as they sought pasture for their livestock. On the other hand, Makueni lies in the arid and semi-arid zones of Eastern Kenya. Horticulture is listed as one of the major economic activities in this area [35]. Thus, victims may have been bitten as they tended to their crops. Snakebite victims were most likely to present to the hospital anywhere between 2 and 6 hours after being bitten by a snake. Similar observations were reported by other workers [26,36]. The culture of the people within the hospital catchment areas was identified as a limiting factor in snake bite management on account of the fact that traditional healers were consulted first before victims visited the health facilities. Belief in traditional healers is entrenched in this part of the world and may explain the low utility of antivenom in some facilities [37]. Additionally, poor accessibility to antivenom, high costs and lack of proper training on management of snake bites may also have contributed to this observation. There was a high uptake of antivenom in areas with a low frequency of snake bites and low uptake of antivenom in areas with high numbers of snake bites. This may stem from the lack of clinical guidelines on the management of snake bites in Kenya. It was also a consensus among the clinicians that no progression of clinical symptoms 24 hours after a snake bite was a key factor in snake bite management using antivenom. This may be a dangerous clinical course as the evolution of symptoms of snake bite is often generally dramatic, differing from patient to patient. Also, the unusually long time taken to evaluate the clinical status of patients may result in complications.

Supportive care coupled with antivenom use was considered most effective in managing snake bites. Supportive care involved wound disinfection, antibiotic use, antihistamines and a tetanus injection. This bodes well with known protocols of snake bite management [38]. However, the efficiency of treatment may be negated by poor supply of antivenom in the health facilities, low demand for antivenom and high costs of antivenom in privately owned pharmacies. However, the problem of inadequate supply of antivenom may not be unique to Kenya. Williamson and colleagues have reported the challenges faced by developing countries in delivering basic medical products such as antivenom [39]. Clinicians in the study area held the belief that available antivenoms were efficacious based on their experience of using them. However, it may not always be possible to vouch for the pharmacological efficacy of antivenom available in hospital settings [22]. The antivenoms we came across in the study area were manufactured in India and France respectively. The Indian antivenom was produced by Bharat Serums and Vaccines Ltd India, batch number A2608003, expiry date 07/11 while the French antivenom was manufactured by Sanofi Pasteur, batch number D5211, expiry 2/2011. Snakes of medical significance in the study area include the black mamba, puff adder, Jameson's mamba and Forest cobra among others [6] (Table 1). The list of snakes on Table 1 may not be exhaustive as there may be a great diversity of snake species in Kenya and some are yet to be identified. Nonetheless, on account of the high survival rate, low uptake of antivenom and low mortality rate observed in our study, it may be suggested that victims may have been subject to “dry bites” or may have been bitten by non-venomous snakes. According to Stewart [40], snake bites may not always be accompanied by envenomation. The mortality rate we observed was much lower than previous reports from other parts of the world [22,41]. It may be difficult to interpret this finding without more information on the actual incidence and outcomes of snake bite in the study area. This study was not without limitations, some of which may affect the extrapolation and interpretation of the data collected. First, victims who may have sought treatment outside the formal healthcare system and did not report bites to our study sites were not captured. This also applies to victims who may have sought care outside the catchment area of our study. Thus, the incidence we have reported may most likely be an underestimate of the true incidence of snake bites in the areas. Secondly, the retrospective nature of our study did not allow for the identification of the offending snakes. Finally, clinical signs of envenomation, adverse effects of antivenom, traditional therapies advanced by herbalists in snake bite management and inventory of antivenom were not reported. The findings of this research can, therefore, be generalized only within these limitations. However, we hold the belief that our facility based study has provided useful insights on factors associated with snake bites in selected areas of Kenya.

## Conclusion

These findings suggest that there is low reporting of snake bites in Kenya. Furthermore, it may be suggested that children and the youth are vulnerable groups. The poor supply and utility of antivenom, little or no effort to identify offending snakes and lack of clinical guidelines on the management of snake bites may jeopardize the management of snake bites in Kenya. There is a need for public awareness of risk factors and first aid management of snake bites. The continuous medical education of healthcare personnel on the management of snake bites and adequate supply of antivenom may improve outcomes of snake bites. Further work on incidence and mortality due to snake bites and animal epidemiological surveys on the distribution of venomous and non-venomous snakes in the country may be important in guiding future public health intervention programs.

### What is known about this topic

- There is poor documentation of snake bite cases in Kenya and reported incidences may be an underestimate of the true burden of snake bites in the country;
- Treatment outcomes of snake bites are relatively good despite snakebite injuries in some areas of Kenya being characterized by compartment syndrome and focal gangrene;
- There is no antivenom that is pre-clinically effective against all the snakes of medical importance in Kenya.

### What this study adds

- Children and young adults should be educated on the importance of protective wear such as shoes while walking and working on farms; moreover, these groups should be educated on preventive measures and first aid treatment of snake bite;
- Policies aimed at improving the supply of antivenom in Kenya are long overdue; there needs to be a structured conversation and political will aimed at solving the problem of snake bite;
- There is a need for clinical guidelines on the management of bites from medically important snakes in Kenya.

## Competing interests

The authors declare no competing interest.
